# Proteomic Analysis Reveals Different Involvement of Embryo and Endosperm Proteins during Aging of Yliangyou 2 Hybrid Rice Seeds

**DOI:** 10.3389/fpls.2016.01394

**Published:** 2016-09-21

**Authors:** Ying-Xue Zhang, Heng-Heng Xu, Shu-Jun Liu, Ni Li, Wei-Qing Wang, Ian M. Møller, Song-Quan Song

**Affiliations:** ^1^Key Laboratory of Plant Resources, Institute of Botany, Chinese Academy of SciencesBeijing, China; ^2^Hunan Hybrid Rice Research Center/State Key Laboratory of Hybrid RiceChangsha, China; ^3^Department of Molecular Biology and Genetics, Aarhus UniversityFlakkebjerg, Denmark

**Keywords:** Yliangyou 2 hybrid rice, proteome, embryo, endosperm, seed aging

## Abstract

Seed aging is a process that results in a delayed germination, a decreased germination percentage, and finally a total loss of seed viability. However, the mechanism of seed aging is poorly understood. In the present study, Yliangyou 2 hybrid rice (*Oryza sativa* L.) seeds were artificially aged at 100% relative humidity and 40°C, and the effect of artificial aging on germination, germination time course and the change in protein profiles of embryo and endosperm was studied to understand the molecular mechanism behind seed aging. With an increasing duration of artificial aging, the germination percentage and germination rate of hybrid rice seeds decreased. By comparing the protein profiles from the seeds aged for 0, 10 and 25 days, a total of 91 and 100 protein spots were found to show a significant change of more than 2-fold (*P* < 0.05) in abundance, and 71 and 79 protein spots were identified, in embryos and endosperms, respectively. The great majority of these proteins increased in abundance in embryos (95%) and decreased in abundance in endosperms (99%). In embryos, most of the identified proteins were associated with energy (30%), with cell defense and rescue (28%), and with storage protein (18%). In endosperms, most of the identified proteins were involved in metabolism (37%), in energy (27%), and in protein synthesis and destination (11%). The most marked change was the increased abundance of many glycolytic enzymes together with the two fermentation enzymes pyruvate decarboxylase and alcohol dehydrogenase in the embryos during aging. We hypothesize that the decreased viability of hybrid rice seeds during artificial aging is caused by the development of hypoxic conditions in the embryos followed by ethanol accumulation.

## Introduction

There are currently more than 7.4 million accessions of seed germplasm conserved in 1750 genebanks around the world, and more than 130 genebanks have 10,000 or more accessions (FAO, 2010). One of the earliest symptoms of seed aging is a delay in radical emergence, followed by a progressive loss of capacity for normal germination (Priestley, 1986; Bewley et al., 2013). This seriously influences the maintenance of seed vigor and the long-term conservation of plant germplasm resource and has led to serious economic losses for agriculture, forestry and horticulture.

Seed aging is affected by genetic components and by storage conditions. Both temperature and seed moisture content are important factors modulating the seed aging rate (Walters et al., 2005; Bewley et al., 2013). Artificial aging, i.e., storing seeds at high temperature and high relative humidity, can mimic natural aging to study seed longevity and vigor (Tesnier et al., 2002; Rajjou et al., 2008; Nguyen et al., 2015; Yin et al., 2015). Reactive oxygen species (ROS) and lipid peroxidation are thought to be the major contributors to seed aging (decrease in vigor), which include loss of membrane integrity, reduction in energy metabolism, decrease in antioxidant system activity, impairment of RNA and protein synthesis, and DNA degradation (Hendry, 1993; Bailly et al., 1996; Bailly, 2004; Bewley et al., 2013; Yin et al., 2014; Xia et al., 2015).

Proteomic approaches are an important tool for determining the biological roles and functions of individual proteins and identifying the molecular mechanisms that govern seed germination and vigor (Rajjou et al., 2008; Wang et al., 2015; Zhang et al., 2015). Proteomic analyses associated with seed aging (vigor change) have been performed on several species, including alfalfa (Yacoubi et al., 2011, 2013), *Arabidopsis thaliana* (Rajjou et al., 2008; Nguyen et al., 2015), *Brassica napus* (Yin et al., 2015), maize (Wu et al., 2011; Xin et al., 2011), poplar (Zhang et al., 2015), sacred lotus (Chu et al., 2012), soybean (Wang et al., 2012a), and sugarbeet (Catusse et al., 2008, 2011). Many different proteins have been proposed to be involved in seed aging, for example, the proteins associated with metabolism, energy, cell growth and division, protein synthesis and destination, storage protein, as well as cell defense and rescue (Rajjou et al., 2008; Catusse et al., 2011; Wu et al., 2011; Xin et al., 2011; Yacoubi et al., 2011; Chu et al., 2012; Wang et al., 2012a; Nguyen et al., 2015; Yin et al., 2015; Zhang et al., 2015). Despite the importance of rice as a model plant and as a crop, the molecular mechanism of seed aging is still poorly understood in this species. The embryo and endosperm are two distinct but interconnected seed components, but their relative contribution is not known in aging seed. In most of the above studies, whole seeds or excised embryos were used as experimental material. Important proteins associated with seed aging might not be detected in embryos when whole seeds are sampled because of the large size of the endosperm, while the role of the endosperm proteins is obviously not monitored when only the embryo is sampled.

The three- and two-line hybrid rice breeding technologies take advantage of heterosis (hybrid vigor) and have been successfully applied in many countries, leading to a more than 20% yield increase over inbred varieties (Cheng et al., 2007). Yliangyou 2 is a super-hybrid rice, which has a super high yield, good plant architecture, high-yielding capacity, fine grain quality, strong stress resistance and wide adaptability (Wu et al., 2015). We observed that the germination of Yliangyou 2 hybrid rice seeds stored for one year at ambient environment at Changsha, China, was less than 80%. However, the reason why seed germination is decreased by storage (aging) is unclear. In the present study, Yliangyou 2 hybrid rice seeds were used to investigate the effect of artificial aging on germination, germination time course and the change in protein profiles of embryo and endosperm during seed aging and in this way provide new knowledge to improve and maintain seed quality.

## Materials and methods

### Ethics statement

No specific permits were required for the described field studies. The location is not privately owned or protected in any way, and the field studies did not involve endangered or protected species.

### Plant materials

Yliangyou 2 (Y58S × Yuanhui 2) hybrid rice (*Oryza sativa* L.) seeds were a generous gift from Hunan Hybrid Rice Research Center (Changsha, Hunan, China). Water content and germination of seeds were 10.7 ± 0.1% (on a fresh weight basis) and 87%, respectively. The seeds were stored in paper bags at −20°C for up to one year without detrimental effects.

### Determination of seed water content

The water content of seeds was gravimetrically determined (at 80°C for 48 h). Four replicates of 25 seeds each were used for determination of water content, and the water content of seeds is expressed on a basis of fresh weight.

### Artificial aging of seeds

To obtain different aging (vigor) levels of seeds, three replicates of 2000 hybrid rice seeds each were stored (aged) in a closed container with 100% relative humidity (RH) and at 40°C for 0, 5, 10, 15, 20, and 25 days, respectively. Water content of seeds increased during artificial aging, and that of seeds artificially aged for 0, 5, 10, 15, 20, and 25 days was 10.7 ± 0.1%, 13.9 ± 0.1%, 14.1 ± 0.0%, 15.1 ± 0.2%, 15.7 ± 0.4%, and 15.9 ± 0.3%, respectively. To decrease the effect of dehydration on the physiological state of the seeds, they were not dried back to the original water content. The seeds were immediately sampled for germination and proteomic analysis after aging.

### Germination testing

Three replicates of 50 seeds each were germinated on two layers of filter paper moistened with 4 ml of MilliQ water in closed 90-mm diameter Petri dishes at 25°C in darkness for 168 h. Germination of seeds was checked every 12 h, and the protrusion of a 2 mm radicle was used as the criterion for completion of germination.

### Preparation of protein samples

The seeds aged for 0, 10, and 25 days, respectively, were manually dehulled and carefully separated into embryo and endosperm, which were immediately frozen in liquid nitrogen. After that, 100 embryos or endosperms were ground to a fine powder in liquid nitrogen with mortar and pestle, and the powder was then kept at −80°C until used.

For extraction of soluble proteins, three replicates of about 0.1 g embryo or 0.75 g endosperm powder each were homogenized in 1.5 ml of precooled extraction buffer contained 50 mM Tris-HCl (pH 7.5), 30% (w/v) sucrose, 10 mM ethylene glycol-bis (β-aminoethylether)- N,N,N′,N′-tetraacetic acid, 1 mM phenylmethylsulfonyl fluoride, 1 mM dithiothreitol (DTT), and 1% (v/v) Triton X-100. After washing the mortar in 0.5 ml extraction buffer, the total of 2 ml homogenate was mixed by vortexing and centrifuged at 4°C at 16,000 g for 10 min, and the supernatant was then centrifuged at 4°C at 32,000 g for 20 min. The resultant supernatant was mixed with two volumes of ice-cold 50 mM Tris-HCl (pH 7.5)-saturated phenol and shaken on ice for 30 min. After centrifugation at 16,000 g for 20 min, the phenol phase was collected and five volumes of precooled methanol saturated with (NH_4_)_2_SO_4_ was added to precipitate the proteins by an overnight incubation at −20°C. The pellets were rinsed four times in ice-cold acetone containing 13 mM DTT, and then lyophilized.

The protein concentration was assayed as described by Bradford (1976) using bovine serum albumin as the standard.

### Two-dimentional (2-D) gel electrophoresis

Isoelectrofocusing (IEF) was performed using a Multiphor II horizontal electrophoresis system (Bio-Rad, Hercules, CA, USA) and 17 cm Immobiline Dry Strips with a linear pH gradient of 5–8 (Bio-Rad). Protein sample was loaded onto the strip soaked in re-hydration solution composed of 7 M urea, 2 M thiourea, 4% (w/v) CHAPS, 20 mM DTT, and 0.5% (v/v) immobilized pH gradient buffer (pH 5–8) at 20°C for 12 h. IEF was then performed by applying a voltage of 250 V for 1 h, ramping to 500 V over 1 h, 2000 V for 2 h, and finally 10,000 V until a total of 60 kVh was reached. Prior to the second dimension, the gel strips were equilibrated for 15 min in equilibration buffer containing 50 mM Tris-HCl (pH 8.8), 6 M urea, 30% (v/v) glycerol, 2% (w/v) SDS, 2% (w/v) DTT or 2.5% (w/v) iodoacetamide. After equilibration, the strips were applied to vertical SDS-polyacrylamide gels (5% stacking and 12% resolving), the low-molecular-range markers (Bio-Rad) were loaded at one end of the strip, and then they were sealed with 0.5% (w/v) low-melting agarose in SDS buffer contained 0.01% (w/v) bromophenol blue. After solidification of the agarose, electrophoresis was performed at 15°C in SDS electrophoresis buffer (pH 8.3) composed of 25 mM Tris base, 192 mM glycine and 1% (w/v) SDS, at 25 mA for 30 min and at 40 mA for 4 h. The gels were stained overnight with 0.25% (w/v) Coomassie brilliant blue R-250 (CBB) in 5:1:4 (v/v) methanol: acetic acid: water, and destained with 2:1:7 (v/v) methanol: acetic acid: water solution with several changes, until a colorless background was achieved.

### Image analysis, in-gel digestion with trypsin and protein identification by MALDI-TOF-TOF mass spectrometry

The 2-D gels were scanned at a 300 dpi resolution in a UMAX Power Look 2100XL scanner (Maxium Tech., Taipei, China). Spot detection and gel comparison were made with ImageMaster 2D Platium (version 5.01; GE Healthcare Bio-Science, Little Chalfont, UK). After automated detection and matching, manual editing was carried out to correct the mismatched and unmatched spots.

Three well-resolved gels of each sample were used to create “replicate groups.” The spots, which were well resolved in all three biological replicates, were considered as reproducible (Supplementary Figures S1, S2). The normalized volume of each spot was assumed to represent the abundance of the detected protein. A criterion of ≥2-fold change (2-fold increase/decrease, *P* < 0.05) was used to define significant differences when comparing spot size among groups.

Protein spots changing in volume were excised from the stained gels. In-gel digestion and tryptic peptide extraction were performed according to the following protocol. Excised spots were washed and destained using a series of washes consisting of 100 μl of water, 100 μl of 50% (v/v) acetonitrile (Fisher Scientific; Fair Lawn, NJ, USA) and 50 μl of 100% acetonitrile. The proteins in the gel pieces were reduced with 10 mM DTT in 100 mM NH_4_HCO_3_ at 56°C for 45 min, and then incubated with 55 mM iodoacetamide in 100 mM NH_4_HCO_3_ at 20°C in darkness for 30 min. After removing iodoacetamide and a series of washes described above, the gel pieces were rehydrated in 50 mM NH_4_HCO_3_ with 10 ng trypsin (sequencing grade modified, Promega, Madison, WI, USA) on ice for 45 min and then incubated overnight at 37°C. After digestion, the supernatant from each sample was recovered and the remaining peptides were then extracted using 5 μl of 5% (v/v) trifluoroactic acid (TFA) followed by 50 μl of 50% (v/v) acetonitrile with 2.5% (v/v) TFA. Each sample was sonicated for 5 min before collecting the supernatant. All supernatants were combined from each spot. After desalting using a column of POROS R2 resin, the samples were spotted on a MALDI target plate, and immediately spotted on top with 0.5 μl of saturated matrix (containing 10 mg/ml α-cyano-4-hydroxycinnamic acid (Sigma), 50% (v/v) acetonitrile, 0.1% (v/v) TFA and 1 mM ammonium phosphate), and dried completely. Samples were then subjected to MALDI-TOF/TOF MS analysis (UltrafleXtreme, Bruker Daltonics, Bremen, Germany).

The peptide mass fingerprints obtained were searched against *Oryza sativa* in the SwissProt and/or NCBI database using MASCOT software (Matrix Science, London, UK). The following search parameters were applied: SwissProt and/or NCBI were used as the protein sequence database; a mass tolerance of 70 ppm in MS mode and 0.5 Da for MS/MS and one incomplete cleavage were allowed; acetylation of the N-terminus, alkylation of cysteine by carbamidomethylation, and oxidation of methionine were considered as possible modifications. The proteins had to meet the following criteria: (1) the probability-based MOWSE score of identified proteins was greater than 48 (*P* < 0.05) in SwissProt database and it was greater than 64 (*P* < 0.05) in NCBI database; (2) the number of matched peptides was at least two.

### Statistical analysis

All data were analyzed with SPSS for Windows 19.0 (SPSS Inc., 2010). Changes in seed germination and accumulation ratios and associated *P*-values for differentially changed proteins in embryos and endosperms during artificial aging of Yliangyou 2 hybrid rice seeds were analyzed using a one-way ANOVA followed by Student-Newman-Keuls multiple comparisons test (S-N-K, *P* = 0.05). All values are expressed as means ± SE (seed germination) or means ± SD (the volume of differentially changed spots).

## Results

### Change in seed viability during artificial aging

**Figure 1:**
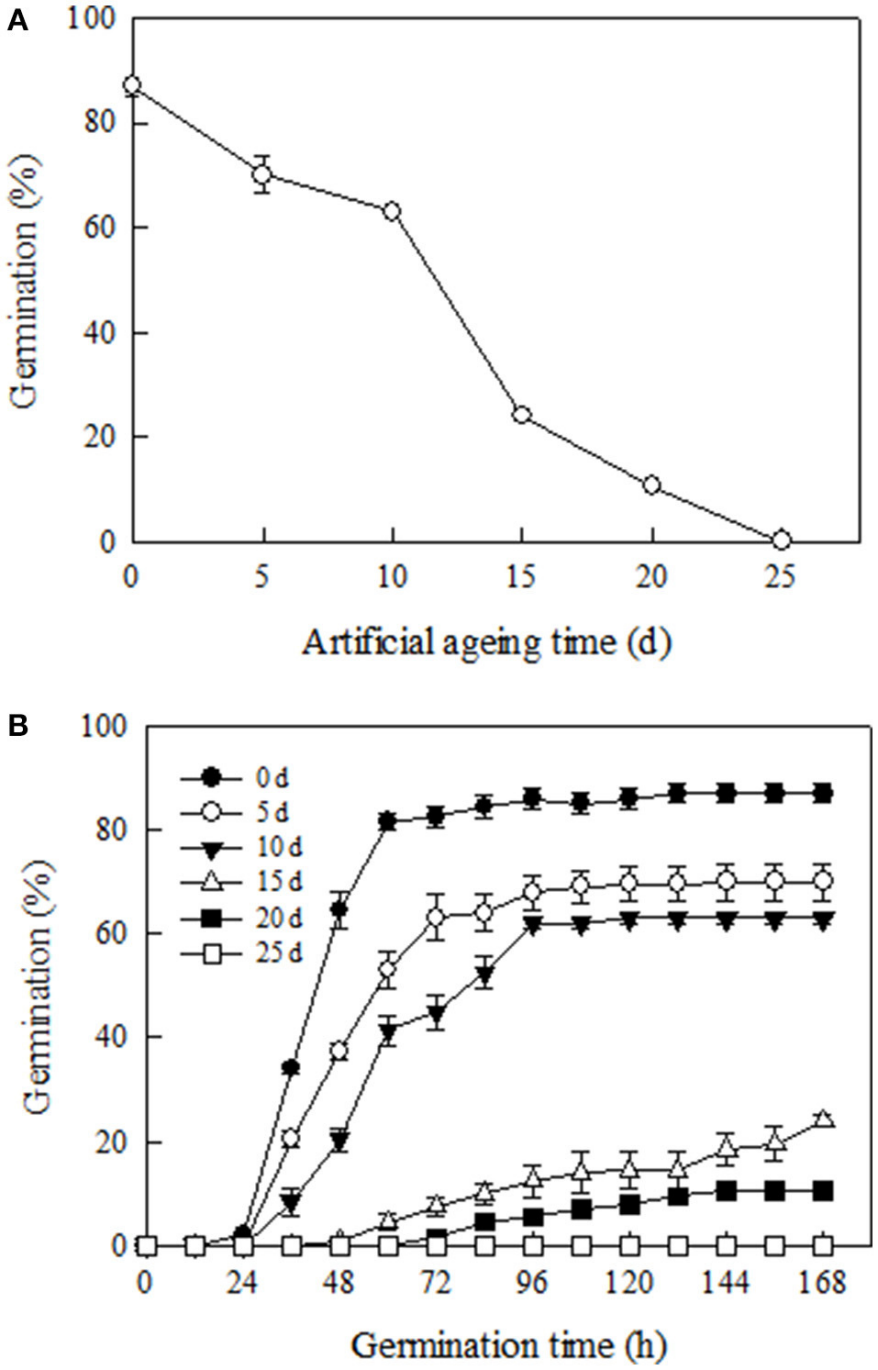
**Changes in (A) germination and (B) germination time course of Yliangyou 2 hybrid rice seeds aged in 100% relative humidity and at 40°C for different periods of time**. After aging for 0, 5, 10, 15, 20, and 25 days, respectively, seeds were germinated at 25°C and in darkness for 168 h. The protrusion of a 2 mm radicle was used as the criterion for completion of germination. All values are means ± SE of three replicates of 50 seeds each.

Germination of Yliangyou 2 hybrid rice seeds, an important parameter to assess seed viability, significantly decreased during aging at 100% RH and 40°C. After 12 days, 50% of the seeds had lost germinability, while none of the seeds aged for 25 days were able to germinate (*P* ≤ 0.001, Figure 1A). We observed that with increasing aging time, the seed germination rate, another important parameter to assess seed viability, also decreased. For example, the time taken to reach 50% germination was about 42, 56 and 80 h, respectively, for seeds aged for 0, 5, and 10 days, and 50% germination was never reached for seeds aged for 15 and 20 days (Figure 1B).

### The proteome profiles, identification and functional classification of proteins changing in abundance

**Figure 2:**
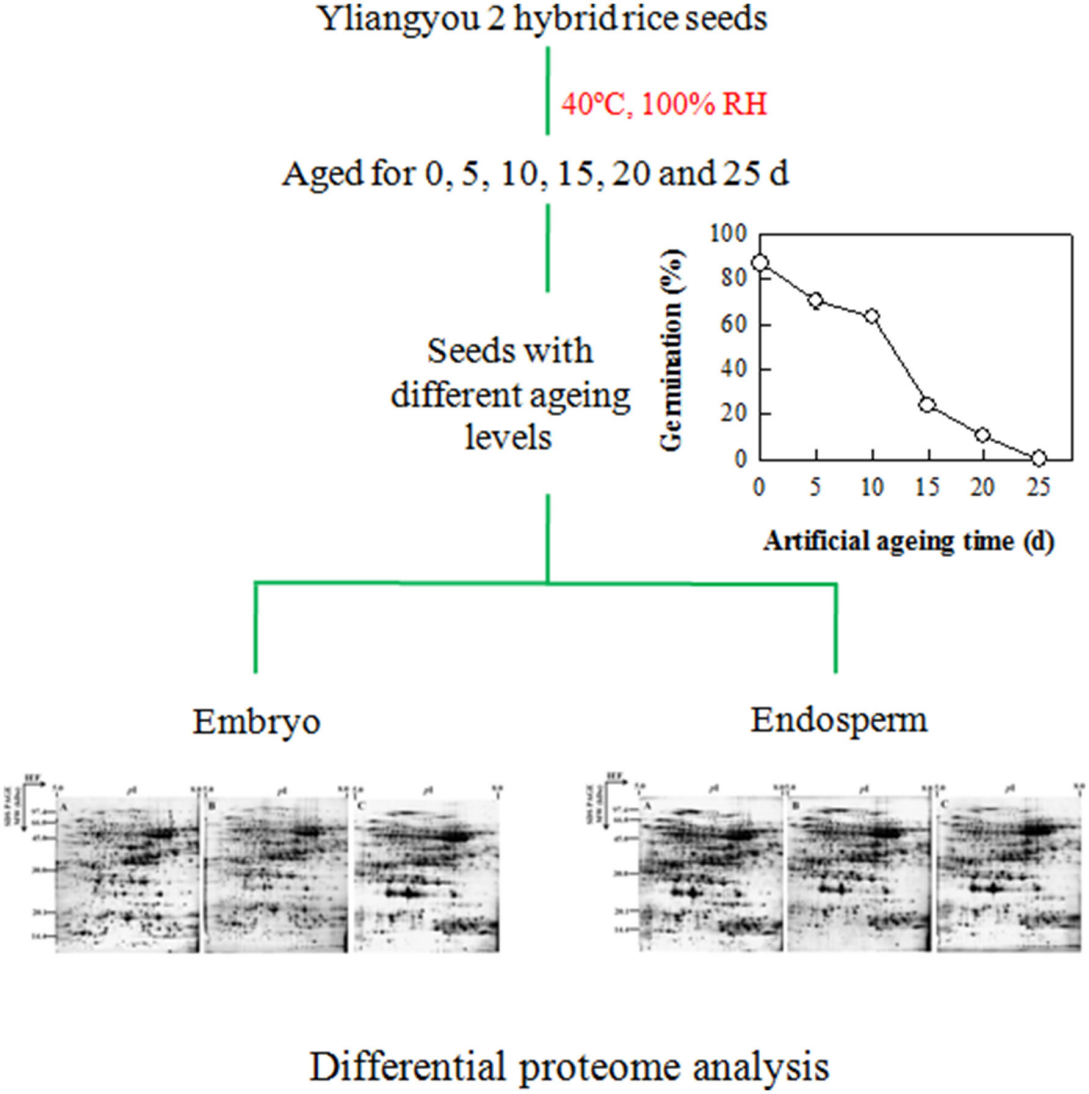
**Schematic drawing of proteomic analysis during aging of Yliangyou 2 hybrid rice seeds**.

**Figure 3:**
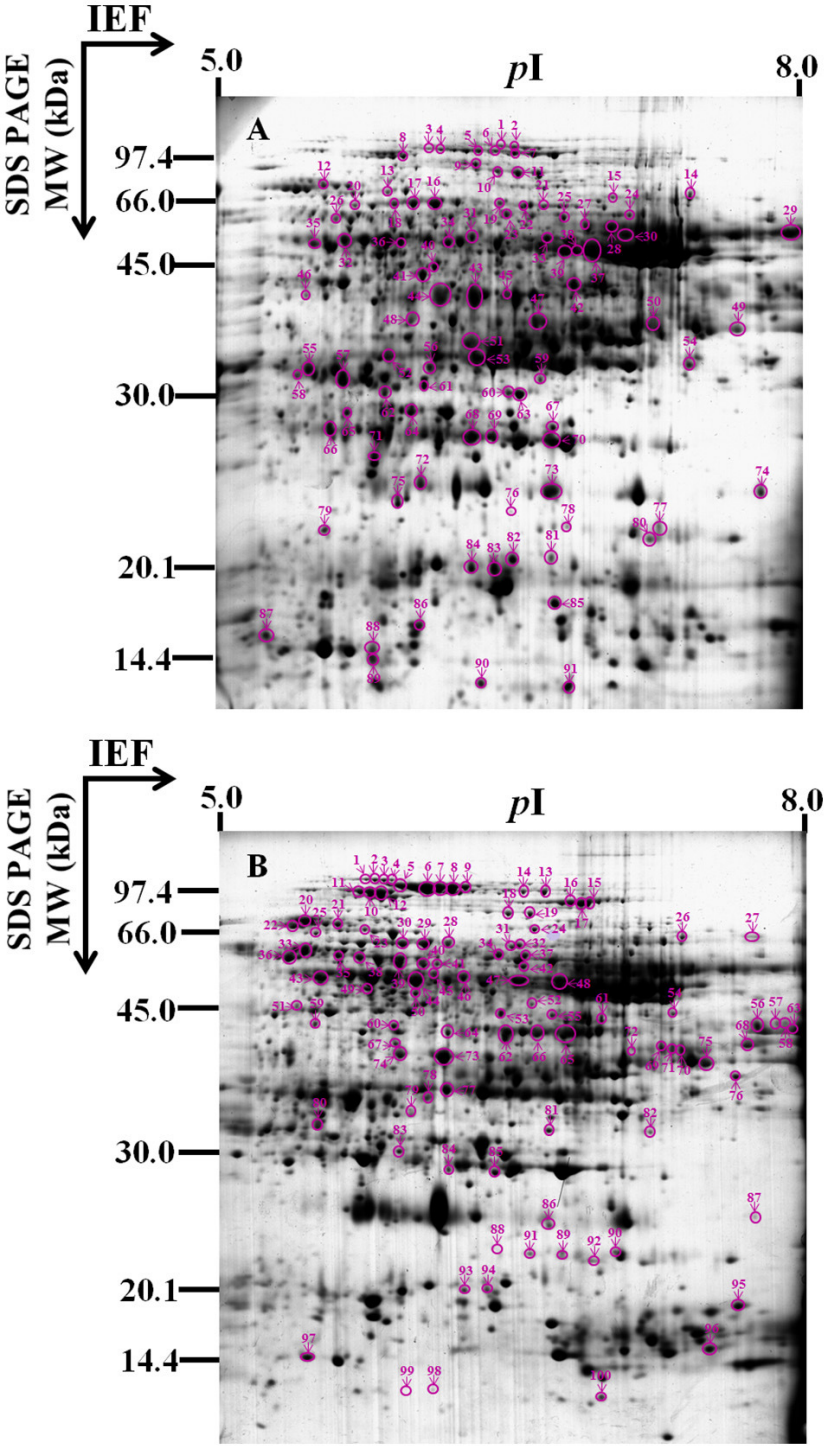
**Gel map of protein spots changing significantly and identified**. This 2-D gel is a representative image of the Commassie Brillant Blue R-250 (CBB) stained gel from **(A)** embryos or **(B)** endosperms of Yliangyou 2 hybrid rice seeds aged in 100% relative humidity and at 40°C for 10 days. A total of 450 μg of proteins from embryos or endosperms of seeds aged for different periods of time was extracted and separated by 2-D gel as described in Materials and methods, and visualized with CBB. The numbered protein spots changed differentially are indicated by arrows and described in Tables 1, 2.

To identify the proteins involved in hybrid rice seed aging, the total protein content of embryos and endosperms from the seeds aged for 0 (A), 10 (B), and 25 (C) days was extracted and analyzed by 2-DE (Figure 2). By comparing the protein profiles of different seed samples, 1109 ± 103 and 1093 ± 93 protein spots were detected in embryo and endosperm, respectively, during aging of Yliangyou 2 hybrid rice seeds (Supplementary Figures S1, S2). Among these protein spots, a total of 91 and 100 protein spots showed a significant change in abundance (≥2-fold increase/decrease, *P* < 0.05) in embryos and endosperms, respectively (Supplementary Tables S1-S4, Figure 3). All of protein spots changing in volume were excised and analyzed by MALDI-TOF/TOF MS, and were identified by first searching in the SwissProt database and, if that showed no matches, then in the NCBI database.

Of 91 protein spots changing in volume in embryos, 71 spots gave one significant protein match, and four spots gave at least two protein matches (Table 1, Supplementary Tables S1, S3, S5, S7, Figure 3A); out of 100 protein spots changing in volume in endosperms, 79 spots gave one significant protein match, and 13 spots gave at least two protein matches (Table 2, Supplementary Tables S2, S4, S6, S8, Figure 3B); when searching in the SwissProt and/or NCBI database. Since we do not know which of the two or more than two proteins showed a significant change in abundance in four protein spots in embryos and 13 protein spots in endosperms among different seed samples (Supplementary Tables S7, S8), these 17 protein spots were not considered in the following analysis.

**Table 1:** **The proteins changing in abundance and identified by MALDI-TOF-TOF MS in embryos during aging of Yliangyou 2 hybrid rice seeds**.

**Biological process**	**Spot ID**	**Identified protein**	**Accession No**.	**Mascot score**	**Sequence coverage (%)**	**No. of sequenced/matched peptides**	**Theo. protein mass (kDa)/pI**	**Exp. protein mass (kDa)/pI**
**METABOLISM (6)**								
Amino acid	10	5-Methyltetrahydropteroyltriglutamate-homocysteine methyltransferase 1^a^	Q2QLY5	479	20	7/13	84.874/5.93	70/6.4
	11	5-Methyltetrahydropteroyltriglutamate-homocysteine methyltransferase 1^a^	Q2QLY5	374	24	6/16	84.874/5.93	69/6.5
	34	Wheat adenosylhomocysteinase-like protein	AAO72664	1080	54	14/25	53.860/5.62	55/6.2
	40	Hypothetical protein OsI_06236 (fumarylacetoacetase)^*^	EEC72678	299	53	4/18	47.659/5.62	51/6.1
Sugar and polysaccharide	52	Glucose and ribitol dehydrogenase homolog	Q75KH3	252	28	6/9	32.475/5.76	38/5.9
Lipid	22	2-Hydroxyacyl-CoA lyase^a^	Q0JMH0	413	31	7/13	61.187/5.95	62/6.6
**ENERGY (21)**								
Glycolysis	13	Os03g0712700 (phosphoglucomutase)^a^	NP_001051066	239	31	3/16	63.138/5.40	65/5.9
	24	Os11g0148500 (pyruvate kinase 1, cytosolic)	NP_001065749	111	13	3/7	57.740/6.30	60/7.1
	32	Enolase	Q42971	645	46	8/17	48.285/5.41	56/5.7
	41	Phosphoglycerate kinase	ABI74567	491	56	5/19	42.224/5.64	50/6.1
	47	Glyceraldehyde-3-phosphate dehydrogenase 3, cytosolic^a^	Q6K5G8	421	32	4/7	36.716/7.68	42/6.6
	49	Glyceraldehyde-3-phosphate dehydrogenase 3, cytosolic^a^	Q6K5G8	1070	55	10/19	36.716/7.68	42/7.6
	65	Hypothetical protein OsI_04384 (triosephosphate isomerase)	EAY76450	471	46	7/11	27.415/5.39	31/5.7
	66	Triosephosphate isomerase, cytosolic	P48494	485	73	6/15	27.274/5.38	30/5.6
TCA cycle	14	Hypothetical protein OsJ_03416 (malic enzyme, NAD^+^-linked)^a^	EAZ13499	758	27	10/16	63.556/6.50	65/7.4
Respiration	35	ATP synthase subunit beta, mitochondrial^a^	Q01859	390	46	8/19	59.012/5.95	55/5.5
	36	ATP synthase subunit alpha, mitochondrial	P0C520	198	12	3/5	55.624/5.85	55/5.9
Fermentation	16	Pyruvate decarboxylase 2^a^	Q10MW3	393	38	5/18	65.761/5.53	63/6.1
	17	Pyruvate decarboxylase 2^a^	Q10MW3	389	33	4/17	65.761/5.53	63/6.0
	18	Pyruvate decarboxylase 2^a^	Q10MW3	443	15	5/6	65.761/5.53	63/5.9
	20	Pyruvate decarboxylase 2^a^	Q10MW3	142	7	3/3	65.761/5.53	63/5.7
	42	Alcohol dehydrogenase 1	Q75ZX4	458	48	6/17	41.699/6.20	48/6.8
Glyoxylate cycle	4	Putative aconitate hydratase, cytoplasmic	Q6YZX6	875	38	10/34	98.591/5.67	76/6.1
	5	Putative aconitate hydratase, cytoplasmic	Q6YZX6	615	35	9/30	98.591/5.67	75/6.3
	48	Malate dehydrogenase (cytoplasmic, NAD^+^-linked)^a^	Q7XDC8	248	35	5/9	35.888/5.75	42/6.0
Photosynthesis	8	Pyruvate, phosphate dikinase 2	Q75KR1	902	40	12/38	97.232/5.42	73/6.0
	33	Ribulose bisphosphate carboxylase large chain	P0C510	718	47	14/23	53.418/6.22	56/6.7
**CELL GROWTH AND DIVISION (1)**								
	79	Os08g0127900 (putative early embryogenesis protein)	NP_001060907	345	15	4/10	58.352/8.72	21/5.6
**TRANSCRIPTION (3)**								
	46	Hypothetical protein OsI_22334 (methyltransferase)^*^	EAZ00317	237	32	3/7	40.445/5.27	41/5.5
	63	Os01g0728700 (histone acetyltransferase)^*^	NP_001044131	366	62	6/14	27.724/5.98	34/6.5
	81	Hypothetical protein OsI_27689 (glycine-rich 2)^*^	EAZ05473	800	84	8/12	18.782/6.28	19/6.7
**PROTEIN SYNTHESIS AND DESTINATION (7)**								
Protein synthesis	6	Os04g0118400 (elongation factor)^a^	NP_001052057	236	17	4/14	94.939/5.85	75/6.4
	7	Os04g0118400 (elongation factor)^a^	NP_001052057	648	40	11/30	94.939/5.85	74/6.5
	19	Hypothetical protein OsI_08509 (aspartyl-tRNA synthetase)^*^	EEC73805	191	26	4/16	61.437/5.99	63/6.4
	39	Elongation factor 1-gamma 3	Q5Z627	253	16	3/7	47.702/6.10	54/6.8
	84	Eukaryotic translation initiation factor 5A-2^a^	ABF98987	419	51	4/11	17.930/5.87	19/6.3
Protein folding	25	Putative t-complex protein 1 theta chain	BAD45605	785	33	10/17	60.683/6.16	60/6.8
Proteolysis	67	Os05g0187000 (proteasome subunit beta type)	NP_001054834	348	20	3/5	29.264/6.45	30/6.7
**STORAGE PROTEIN (13)**								
	29	Putative globulin (with alternative splicing)^a^	AAS07324	1040	46	12/21	63.845/8.35	57/7.9
	30	Putative globulin (with alternative splicing)^a^	AAS07324	816	46	10/21	63.845/8.35	56/7.1
	38	Hypothetical protein OsI_13867 (globulin-like protein)^*^	EEC76319	706	48	9/23	52.370/6.99	54/6.8
	51	Hypothetical protein OsI_13867 (globulin-like protein)^*^	EEC76319	932	34	10/17	52.370/6.99	40/6.3
	53	Hypothetical protein OsI_13867 (globulin-like protein)^*^	EEC76319	301	18	3/6	52.370/6.99	38/6.3
	56	Putative globulin (with alternative splicing)	AAS07324	330	27	5/10	63.845/8.35	36/6.1
	57	Os03g0663800 (putative globulin, with alternative splicing)^*^	NP_001173574	248	15	3/6	45.512/6.07	35/5.7
	58	Os03g0663800 (putative globulin, with alternative splicing)^*^	NP_001173574	222	22	4/7	45.512/6.07	36/5.4
	61	Putative globulin (with alternative splicing)^a^	AAS07324	439	29	6/11	63.845/8.35	34/6.1
	62	Cupin family protein, expressed	ABF95817	195	20	4/9	61.742/7.18	34/5.9
	64	Hypothetical protein OsI_13867 (globulin-like protein)^*^	EEC76319	466	28	4/16	52.370/6.99	31/6.0
	71	Globulin-like protein^a^	AAM33459	294	25	3/13	52.376/6.78	27/5.8
	75	Globulin-like protein^a^	AAM33459	761	23	7/13	52.376/6.78	23/5.9
**CELL DEFENSE AND RESCUE (20)**								
Defense-related	69	Cysteine proteinase inhibitor 12	Q0JNR2	239	50	3/13	27.252/6.07	29/6.4
	83	Bowman-Birk type bran trypsin inhibitor	A2WK50	65	12	3/4	29.219/5.38	19/6.4
Detoxifacation	31	Aldehyde dehydrogenase	AAF73828	927	32	10/19	59.626/6.33	56/6.3
	54	Hypothetical protein OsI_08976 (annexin)	EAY87564	548	64	9/23	35.689/7.13	37/7.4
	55	Lactoylglutathione lyase	Q948T6	716	39	9/13	32.875/5.51	36/5.5
	60	Lactoylglutathione lyase	Q948T6	103	10	2/4	32.875/5.51	35/6.5
	68	1-Cys peroxiredoxin A	P0C5C9	237	56	4/11	24.198/5.97	29/6.3
	70	1-Cys peroxiredoxin A	P0C5C9	255	58	4/12	24.198/5.97	28/6.7
	82	Superoxide dismutase	A2XGP6	190	20	2/3	15.356/5.71	19/6.5
	85	Superoxide dismutase	P28757	255	48	2/5	15.185/5.92	17/6.7
Stress response	1	Chaperone protein ClpB1	Q6F2Y7	580	30	10/25	101.062/5.90	76/6.4
	2	Chaperone protein ClpB1	Q6F2Y7	1040	43	13/36	101.062/5.90	76/6.5
	12	Heat shock cognate 70 kDa protein, putative, expressed^a^	ABF95267	237	24	4/13	71.932/5.30	67/5.6
	27	Hypothetical protein OsI_06577 (putative late embryogenesis abundant protein)^*^	EEC72841	299	19	4/7	45.019/6.50	58/6.9
	28	Hypothetical protein OsI_06577 (putative late embryogenesis abundant protein)^*^	EEC72841	456	40	6/17	45.019/6.50	58/7.0
	43	Late embryogenesis abundant protein 1	A2XG55	412	55	4/18	35.869/6.01	46/6.3
	44	Late embryogenesis abundant protein 1	A2XG55	227	34	3/9	35.869/6.01	46/6.1
	74	Late embryogenesis abundant protein, group 3^a^	A2Y720	325	28	4/5	20.455/6.45	24/7.8
	76	Os02g0707900 (ethylene-responsive protein-like)	NP_001047879	76	20	2/4	20.204/5.96	23/6.5
	86	Glycine-rich RNA binding protein	ACA50486	229	67	3/11	16.089/6.32	15/6.0

*Only protein spots that changed in volume at least 2-fold in all three replicates for a given treatment are included. Some fold changes are between −2 and +2, because there is a change of at least 2-fold in one of the treatments (see Supplementary Table S3 for spot volume and P-value). The positions of the spots are shown in Figure 3A. No, number; Theo, theoretical; Exp, experimental*.

* *Blast result*.

a *This protein also changed significantly in the endosperm*.

**Table 2:** **The proteins changing in abundance and identified by MALDI-TOF-TOF MS in endosperms during aging of Yliangyou 2 hybrid rice seeds**.

**Biological process**	**Spot ID**	**Identified protein**	**Accession No**.	**Mascot score**	**Sequence coverage (%)**	**No. of sequenced/matched peptides**	**Theo. protein mass (kDa)/pI**	**Exp. protein mass (kDa)/pI**
**METABOLISM (29)**								
Amino acid	18′	5-Methyltetrahydropteroyltriglutamate-homocysteine methyltransferase 1^a^	Q2QLY5	1100	35	14/21	84.874/5.93	76/6.6
	19′	5-Methyltetrahydropteroyltriglutamate-homocysteine methyltransferase 1^a^	Q2QLY5	1070	34	12/24	84.874/5.93	76/6.7
	35′	Ketol-acid reductoisomerase, chloroplastic	Q65XK0	139	17	4/7	62.680/6.01	66/5.7
	38′	Ketol-acid reductoisomerase, chloroplastic	Q65XK0	612	27	7/14	62.680/6.01	65/5.8
	39′	Ketol-acid reductoisomerase, chloroplastic	Q65XK0	818	30	7/18	62.680/6.01	64/6.0
	47′	Os10g0390500 (alanine aminotransferase)	NP_001064504	1030	66	13/36	53.130/6.23	60/6.6
	48′	Putative alanine aminotransferase	AAK52114	1410	57	16/28	53.229/6.23	60/6.8
	52′	γ-Aminobutyrate transaminase 1, mitochondrial	Q01K11	493	40	8/16	56.620/6.33	55/6.7
	56′	Aspartate aminotransferase, cytoplasmic	P37833	352	39	6/15	44.650/7.75	51/7.9
	57′	Aspartate aminotransferase, cytoplasmic	P37833	220	31	4/11	44.650/7.75	52/8.0
	61′	Glutamate dehydrogenase 2	BAE48298	489	45	9/14	44.865/6.21	51/7.1
Sugar and polysaccharide	1′	α-1,4-glucan phosphorylase L isozyme, partial	AAK15695	1050	48	14/36	105.091/5.38	86/5.8
	2′	α-1,4-glucan phosphorylase L isozyme, partial	AAK15695	747	31	12/25	105.091/5.38	86/5.9
	3′	α-1,4-glucan phosphorylase L isozyme, partial	AAK15695	1000	49	14/39	105.091/5.38	86/5.9
	4′	α-1,4-glucan phosphorylase L isozyme, partial	AAK15695	313	15	5/13	105.091/5.38	86/6.0
	5′	Pullulanase	ACY56108	1230	46	15/36	103.079/5.44	84/6.0
	6′	Pullulanase	ACY56108	1490	50	14/39	103.079/5.44	83/6.1
	7′	Pullulanase	ACY56108	1220	46	14/35	103.023/5.58	83/6.2
	8′	Hypothetical protein OsJ_13773 (pullulanase)^*^	EEE60487	1340	38	15/29	100.410/5.58	83/6.3
	9′	Hypothetical protein OsJ_13773 (pullulanase)^*^	EEE60487	973	39	13/30	100.410/5.58	84/6.3
	15′	Sucrose synthase 3	Q43009	888	49	12/42	93.566/6.15	79/7.0
	16′	Sucrose synthase 3	Q43009	840	31	14/26	93.566/6.15	79/6.9
	17′	Sucrose synthase 3	Q43009	885	48	11/40	93.566/6.15	79/6.9
	42′	UDP-glucose pyrophosphorylase	ABD57308	858	61	10/20	51.791/5.43	63/6.6
	44′	UDP-glucose pyrophosphorylase	ABI83672	1490	79	14/33	51.813/5.59	60/6.1
	64′	UDP-arabinopyranose mutase 1	Q8H8T0	668	52	10/20	41.835/5.82	50/6.2
	65′	Hypothetical protein OsI_30129 (sorbitol dehydrogenase)^*^	EEC83982	801	67	9/21	39.886/6.15	49/6.9
Nucleotide	53′	Adenylosuccinate synthetase, chloroplastic	A2XD35	1190	46	13/19	52.546/6.39	53/6.5
Lipid	31′	2-Hydroxyacyl-CoA lyase^a^	Q0JMH0	1010	52	13/26	61.187/5.95	68/6.6
**ENERGY (21)**								
Glycolysis	23′	Os03g0712700 (phosphoglucomutase)^a^	NP_001051066	912	46	14/26	63.138/5.40	72/5.8
	37′	Os06g0247500 (putative pyrophosphate-dependent phosphofructokinase beta subunit)	NP_001057284	993	70	12/31	61.907/6.01	66/6.6
	68′	Fructose-bisphosphate aldolase cytoplasmic isozyme	P17784	583	37	6/10	39.238/6.96	47/7.8
	69′	Fructose-bisphosphate aldolase cytoplasmic isozyme	P17784	138	14	4/4	39.238/6.96	46/7.4
	70′	Os01g0905800 (fructose-bisphosphate aldolase)	NP_001045130	153	27	3/7	39.141/8.35	46/7.5
	71′	Fructose-bisphosphate aldolase cytoplasmic isozyme	P17784	235	15	5/6	39.238/6.96	46/7.5
	75′	Glyceraldehyde-3-phosphate dehydrogenase 3, cytosolic^a^	Q6K5G8	881	56	8/17	36.716/7.68	44/7.6
TCA cycle	26′	Hypothetical protein OsI_03698 (malic enzyme, NAD+-linked)^a^	EAY75782	670	28	12/18	63.578/7.11	62/7.5
	54′	Citrate synthase	AAG28777	512	36	10/15	52.423/7.71	53/7.5
	59′	Succinyl-CoA ligase [ADP-forming] subunit beta, mitochondrial	Q6K9N6	400	23	4/10	45.405/5.98	52/5.6
Respiration	43′	ATP synthase subunit beta, mitochondrial^a^	Q01859	1580	71	15/26	59.012/5.95	61/5.6
Fermentation	28′	Pyruvate decarboxylase 2^a^	Q10MW3	505	34	7/14	65.761/5.53	70/6.2
	29′	Pyruvate decarboxylase 2^a^	Q10MW3	549	35	9/16	65.761/5.53	68/6.1
	30′	Pyruvate decarboxylase 2^a^	Q10MW3	564	31	9/15	65.761/5.53	68/6.0
	55′	Alcohol dehydrogenase 2	Q4R1E8	179	27	4/8	41.978/6.04	53/6.8
Glyoxylate cycle	73′	Malate dehydrogenase (cytoplasmic, NAD+-linked)^a^	Q7XDC8	1240	57	10/13	35.888/5.75	46/6.2
	74′	Malate dehydrogenase (cytoplasmic, NAD+-linked)^a^	Q7XDC8	780	60	9/16	35.888/5.75	46/6.0
Photosynthesis	10′	Pyruvate phosphate dikinase 1, chloroplastic	Q6AVA8	1340	46	14/39	103.578/5.98	82/5.8
	11′	Pyruvate phosphate dikinase 1, chloroplastic	Q6AVA8	1100	40	14/29	103.578/5.98	83/5.8
	12′	Pyruvate phosphate dikinase 1, chloroplastic	Q6AVA8	1310	51	13/42	103.578/5.98	81/5.9
Others	60′	Os04g0386600 (2-methylisocitrate lyase)^*^	NP_001052622	788	51	9/15	41.636/5.66	51/6.0
**CELL GROWTH AND DIVISION (5)**								
	25′	Os06g0662000 (putative vacuolar proton-ATPase)	NP_001058280	1100	52	14/32	68.711/5.20	71/5.6
	51′	Os05g0438800 (actin)	NP_001055661	155	19	3/5	41.866/5.29	55/5.5
	97′	Embryonic abundant protein 1	P46520	146	47	3/5	10.159/5.57	15/5.4
	99′	Early embryogenesis protein	AAD10370	70	4	2/2	45.080/10.54	13/6.2
	100′	Differentiation embryo protein 31	ABC74439	89	5	2/3	49.213/6.04	13/7.1
**PROTEIN SYNTHESIS AND DESTINATION (9)**								
Protein synthesis	13′	Os02g0519900 (elongation factor 2)	NP_001046972	837	43	12/30	94.987/5.85	82/6.8
	14′	Os04g0118400 (elongation factor)^a^	NP_001052057	907	34	12/23	94.939/5.85	82/6.6
	32′	Os02g0686400 (putative aspartate-tRNA ligase)	NP_001047770	634	50	10/29	61.451/5.99	68/6.6
	49′	Eukaryotic initiation factor 4A-1	P35686	571	49	8/22	47.343/5.37	59/5.8
	93′	Eukaryotic translation initiation factor 5A-2, putative, expressed^a^	ABF98987	304	50	3/8	17.930/5.87	20/6.3
Protein folding	36′	Putative rubisco subunit binding-protein alpha subunit precursor	AAP44754	1060	55	13/23	61.477/5.36	66/5.4
Proteolysis	40′	Leucine aminopeptidase 2, chloroplastic	Q6K669	531	31	7/17	62.179/8.29	63/6.1
	83′	Os02g0634500 (ATP-dependent Clp protease proteolytic subunit)^*^	NP_001047512	244	38	4/8	32.112/6.71	32/6.0
Protein transport	50′	Os05g0304400 (GDP dissociation inhibitor protein OsGDI1)^*^	NP_001055142	804	57	9/23	50.074/5.54	57/6.1
**STORAGE PROTEIN (5)**								
	20′	Endosperm lumenal binding protein	AAB63469	1440	37	14/27	73.666/5.30	75/5.5
	77′	Os05g0116000 (putative legumin)	NP_001054469	842	62	7/18	38.456/5.81	40/6.2
	79′	Putative globulin (with alternative splicing)^a^	AAS07324	803	33	10/13	63.845/8.35	37/6.1
	95′	Seed allergenic protein RAG2	Q01882	288	28	4/5	18.423/8.06	18/7.8
	96′	Globulin-like protein^a^	AAM33459	207	17	3/6	52.376/6.78	15/7.6
**CELL DEFENSE AND RESCUE (7)**								
Defense-related	62′	Serpin-ZXA	Q75H81	855	64	8/19	42.114/7.75	49/6.5
Detoxifacation	84′	Os05g0116100 (dehydroascorbate reductase)	NP_001054470	604	71	9/11	23.726/5.81	30/6.2
	85′	Os05g0116100 (dehydroascorbate reductase)	NP_001054470	939	67	9/13	23.726/5.81	30/6.5
Stress response	21′	Heat shock cognate 70 kDa protein, putative, expressed^a^	ABF95267	932	52	13/35	71.932/5.30	74/5.3
	22′	Heat shock protein 70	CAA47948	671	35	10/22	71.352/5.17	74/5.4
	24′	Os02g0644100 (putative stress-induced protein sti1)	NP_001047563	811	47	13/23	65.159/6.03	72/6.7
	86′	Late embryogenesis abundant protein, group 3^a^	P0C5A4	115	27	2/3	20.502/5.89	26/6.8
**UNKNOWN (3)**								
	63′	Hypothetical protein OsI_10172 (embryonic protein DC-8 precursor)^*^	EAY88696	249	23	4/11	39.933/8.29	50/8.1
	76′	Hypothetical protein OsI_27370 (osr40g2)^*^	EAZ05175	830	62	11/18	39.715/7.29	42/7.8
	82′	Os03g0822200 (NAD-dependent epimerase/dehydratase)^*^	NP_001051733	741	60	9/13	27.950/6.34	35/6.8

*Only protein spots that changed in volume at least 2-fold in all three replicates for a given treatment are included. Some fold changes are between −2 and +2, because there is a change of at least 2-fold in one of the treatments (see Supplementary Table S4 for spot volume and P-value). The positions of the spots are shown in Figure 3B. No, number; Theo, theoretical; Exp, experimental*.

* *Blast result*.

a *This protein also changed significantly in the embryo*.

**Figure 4:**
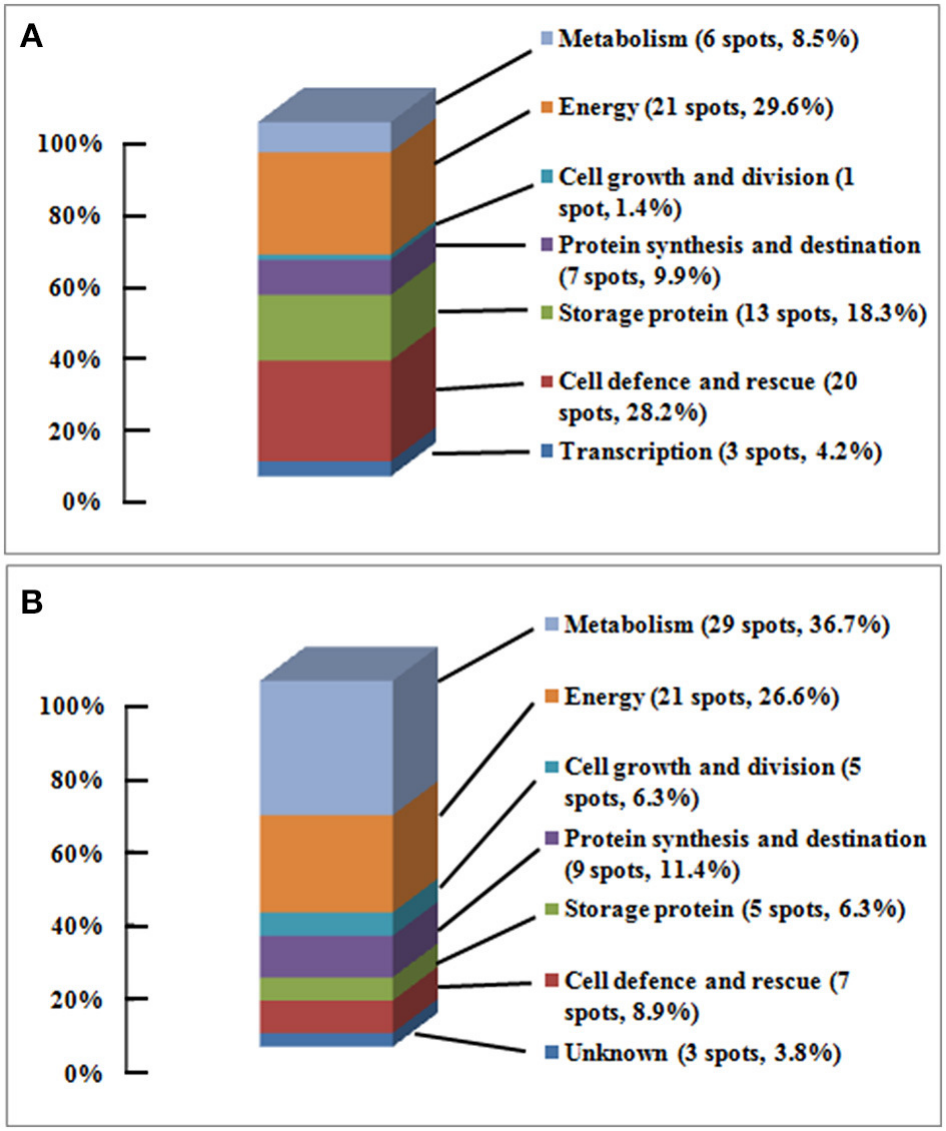
**The functional classification and distribution of proteins changing in abundance and identified. (A)** Embryo, 71 protein spots changed in volume were categorized into 7 functional groups and 17 sub-functional groups; **(B)** Endosperm, 79 protein spots changing in volume were categorized into 7 functional groups and 19 sub-functional groups; based upon Bevan et al. (1998) and Schiltz et al. (2004).

The identified 71 single protein spots in embryos were matched to 51 unique genes, and could be classified into seven functional groups and 18 subfunctional groups based upon Bevan et al. (1998) and Schiltz et al. (2004) (Table 1, Figure 4A). Most of the identified proteins were associated with energy (30%), with cell defense and rescue (28%), with storage protein (18%), with protein synthesis and destination (10%), and with metabolism (9%); these proteins accounted for 95% of all identified proteins in the embryos (Figure 4A). Furthermore, the identified 79 single protein spots in endosperms were matched to 60 unique genes, and could be classified into seven functional groups and 21 subfunctional groups (Table 2, Figure 4B). Of these proteins, 37% protein spots were related to metabolism, 27% to energy, 11% to protein synthesis and destination, 9% to cell defense and rescue, 6% to storage protein, and 6% to cell growth and division; these proteins accounted for 96% of all identified proteins in the endosperms (Table 2, Figure 4B).

### The proteins changing in abundance in embryos and endosperms during seed artificial aging

By comparing the proteome profiles of embryos and endosperms from seeds aged for 0, 10, and 25 days, respectively, the proteins changing in abundance can be classified into those with increased/decreased abundance and those with a complex abundance pattern (Tables 1–3, Supplementary Tables S3, S4, Figure 3). The increased proteins are those whose abundance, compared to 0 day, increased by ≥2-fold at 10 days of aging and stayed high or increased further at 25 days of aging, and those whose abundance showed a less than 2-fold change at 10 days of aging and then a ≥2-fold increase. The decreased proteins are those whose abundance, compared to 0 day, decreased by ≥2-fold at 10 days of aging and stayed low or decreased further at 25 days of aging, and those whose abundance showed a less than 2-fold change at 10 days of aging and then a ≥2-fold decrease. The proteins whose abundance first increased by ≥2-fold and then decreased significantly (including less than 2-fold change) or vice versa during seed aging are considered to show a complex pattern (Tables 1–3, Supplementary Tables S3, S4, Figure 3).

**Table 3:** **The proteins changing in abundance and identified in embryo and endosperm during aging of Yliangyou 2 hybrid rice seeds**.

**Biological process**	**Embryo**				**Endosperm**			
	**Increased**		**Decreased**		**Increased**		**Decreased**	
	**No**.	**Spot ID**	**No**.	**Spot ID**	**No**.	**Spot ID**	**No**.	**Spot ID**
**Metabolism**	**5**						**29**	
Amino acid	4	10, 11, 34, 40					11	18′, 19′, 35′, 38′, 39′, 47′, 48′, 52′, 56′, 57′, 61′
Sugar and polysaccharide	1	52					16	1′, 2′, 3′, 4′, 5′, 6′, 7′, 8′, 9′, 15′, 16′, 17′, 42′, 44′, 64′, 65′,
Nucleotide							1	53 ′
Lipid							1	31′
**Energy**	**19**		**1**				**21**	
Glycolysis	7	13, 24, 32, 47, 49, 65, 66	1	41			7	23′, 37′, 68′, 69′, 70′, 71′, 75′
TCA cycle	1	14					3	26′, 54′, 59′,
Respiration	2	35, 36					1	43′
Fermentation	5	16, 17, 18, 20, 42					4	28′, 29′, 30′, 55′
Glyoxylate cycle	3	4, 5, 48					2	73′, 74′
Photosynthesis	1	33					3	10′, 11′, 12′
Others							1	60′
**Cell growth and division**	**1**	79			**1**	100′	**3**	25′, 51′, 97′
**Transcription**	**2**	46, 63	**1**	81				
**Protein synthesis and destination**	**5**						**8**	
Protein synthesis	4	6, 7, 19, 39					4	13′, 32′, 49′, 93′
Protein folding							1	36′
Proteolysis	1	67					2	40′, 83′
Protein transport							1	50′
**Storage protein**	**11**	29, 30, 38, 51, 53, 56, 57, 58, 64, 71, 75					**5**	20′, 77′, 79′, 95′, 96′
**Cell defense and rescue**	**15**		**1**				**7**	
Defense-related	1	69					1	62′
Detoxification	6	31, 54, 55, 60, 68, 82					2	84′, 85′
Stress response	8	1, 2, 12, 27, 28, 43, 44, 86	1	76			4	21′, 22′, 24′, 86′
**Unknown**							**3**	63′, 76′, 82′
Total	58		3		1		76	

*Only protein spots which changed in volume at least 2-fold (P < 0.05) in all three replicates for a given treatment are included. The positions of the protein spots are shown in Figure 3. The bold values represent the protein spot number of functional group*.

During seed aging, 61 embryo protein spots showed an increase/decrease in volume, and 10 spots showed a complex pattern; while 77 endosperm protein spots showed a decrease/increase in volume, and two spots showed a complex pattern (Table 3, Supplementary Tables S3, S4, Figure 3). Of the 61 embryo protein spots, 58 increased and three decreased in abundance. Out of the increased proteins, 19 were involved in energy, 15 in cell defense and rescue, 11 in storage protein, five in metabolism, five in protein synthesis and destination, two in transcription, and one in cell growth and division. The three embryo proteins decreasing in abundance were associated with energy, transcription and cell defense and rescue (Table 3, Supplementary Table S3, Figure 3A).

Out of 77 changing protein spots in endosperms, 76 decreased in volume while only one increased. Among the proteins decreasing in abundance, 29 were involved in metabolism, 21 in energy, eight in protein synthesis and destination, seven in cell defense and rescue, five in storage protein, three in cell growth and division, and three in unknown protein; for the increased protein, only one protein was implicated in cell growth and division (Table 3, Supplementary Table S4, Figure 3B).

## Discussion

### Changes in viability during artificial aging of hybrid rice seeds

When hybrid rice seeds were exposed to 100% RH at 40°C, germination percentage and germination rate of seeds significantly decreased (Figure 1), indicating that seed viability decreased during artificial aging. These results are similar to those of Rajjou et al. (2008), Xin et al. (2011), Zhang et al. (2015) and Gao et al. (2016), who found that seed viability gradually decreased with increasing aging time. In this way, we obtained seed samples with different viability (aging level).

### Changes in protein profiles of embryos and endosperms during artificial aging of hybrid rice seeds

To understand the action of embryo and endosperm and their interaction in seed aging, the differentially changing proteins in embryo and endosperm were compared in hybrid rice seeds with different aging levels. Using a ≥2.0-fold change (*P* < 0.05) in abundance as the significance level, we found that during aging of hybrid rice seeds, 95% of the changing proteins increased in embryos, while 99% decreased in endosperms (Table 3, Supplementary Tables S3, S4, Figure 3). Most of the differentially changing proteins were embryo- or endosperm-specific, 14 proteins (spots 10, 11/18′, 19′; 22/31′; 13/23′; 47, 49/75′; 14/26′; 35/43′; 16, 17, 18, 20/28′, 29′, 30′; 48/73′, 74′; 6, 7/14′; 84/93′; 29, 30, 61/79′; 71, 75/96′; 12/21′; 74/86′) were commonly present in both embryo and endosperm (Tables 1-3, Supplementary Tables S3, S4, Figure 3), although their abundance changes were opposite. These results indicate that the embryo and endosperm proteomes were differentially affected by aging.

It is possible that seed aging is an active process, in which the embryo tries to adjust to the aging conditions by synthesizing a range of proteins, while the endosperm provides amino acids and related products for protein synthesis in the embryo by degrading proteins. The endosperm is considered to be a “dead” tissue in the sense that, except for the aleurone layer, there are no cells that can grow and divide (Bewley et al., 2013). Any change in the starchy endosperm dependent on changes in gene expression therefore requires some form of communication with the aleurone layer and/or the embryo. The induction of α-amylase biosynthesis in the aleurone layer by gibberellic acid produced in the embryo during the early stages of germination is an example of this (Bewley et al., 2013). Han et al. (2014) reported a much broader regulation of endosperm metabolism by the embryo in germinating rice seeds. Our results indicate that a similar regulation of endosperm metabolism by the embryo could take place during aging of rice seeds.

#### Proteins associated with metabolism

##### Proteins involved in amino acid metabolism increased in embryos and decreased in endosperms

Four protein spots increased in embryos, and 11 protein spots decreased in endosperms (Table 3, Figure 3). 5-Methyltetrahydropteroyltriglutamate-homocysteine S-methyltransferase (also known as methionine synthase), which is a housekeeping enzyme, catalyzes the last step in the methionine biosynthetic pathway in plant. Adenosylhomocysteinase (AdoHcyase) catalyzes the conversion of S-adenosylhomocysteine to homocysteine and adenosine in the methyl cycle (Gallardo et al., 2002; Rajjou et al., 2012). Methionine synthase 1 (spots 10 and 11) and wheat adenosylhomocysteinase-like protein (spot 34) abundance increased in embryos, and methionine synthase 1 (spots 18′ and 19′) decreased in endosperms (Table 3, Supplementary Tables S3, S4, Figure 3), indicating that these proteins are implicated in hybrid rice seed aging, but have different roles in embryos and endosperms. Catusse et al. (2011) proposed that the active methyl cycle play an important role in seed vigor.

Fumarylacetoacetase, which is also known as fumarylacetoacetate hydrolase, catalyzes the hydrolytic cleavage of a carbon-carbon bond in fumarylacetoacetate to yield fumarate and acetoacetate as the final step in phenylalanine and tyrosine degradation (Nelson and Cox, 2005). Hypothetical protein OsI_06236 (fumarylacetoacetase, spot 40) increased in embryos (Table 3, Supplementary Table S3, Figure 3A), indicating that this protein is implicated in hybrid rice seed aging. Han et al. (2013) demonstrated that disruption of fumarylacetoacetase led to call death in Arabidopsis and suggested that the tyrosine degradation pathway was essential for plant survival under short-day conditions.

Alanine aminotransferase (AlaAT), a pyridoxal-5′-phosphate-dependent enzyme, catalyzes the reversible transfer of an amino group from alanine to 2-oxoglutarate to form glutamate and pyruvate. AlaAT is implicated in numerous cellular processes including glycolysis, gluconeogenesis, amino acid metabolism, photorespiration and nitrogen use efficiency (McAllister et al., 2013). Aspartate aminotransferase (AspAT) catalyzes the reversible transamination between aspartate and 2-oxoglutarate to yield glutamate and oxaloacetate, and plays a key role in carbon and nitrogen distribution in plant (Lam et al., 1995). Glutamate dehydrogenase (GluDH) converts ammonium and 2-oxoglutarate to glutamate and plays a key role in nitrogen and glutamate metabolism (Taiz et al., 2014). Ketol-acid reductoisomerase (KARI) is an enzyme in the biosynthesis pathway of branched-chain amino acid where it catalyzes the conversion of 2-acetolactate into (2*R*)-2,3-dihydroxy-3-isovalerate or the conversion of 2-aceto-2-hydroxybutyrate into (2*R*,3*R*)-2,3-dihydroxy-3-methylvalerate (Leung and Guddat, 2009). AlaAT (spots 47′ and 48′), AspAT (spots 56′ and 57′), GluDH 2 (spot 61′) and KARI (spots 35′, 38′, and 39′) abundances all decreased in the endosperms (Table 3, Supplementary Table S4, Figure 3B). This decline may lead to a decreased supply of amino acids to the embryo thereby contributing to hybrid rice seed aging.

##### Proteins involved in sugar and polysaccharide metabolism decreased in abundance in the endosperm

α-1,4-Glucan phosphorylases are pyridoxal 5′-phosphate-dependent glucosyltransferases, and catalyze the conversion of oligo- and polyglucosidic substrates into α-D-glucose 1-phosphate, thereby fueling the energy metabolism of the cell (Mueller et al., 2009). Sucrose synthase catalyzes the UDP-dependent cleavage of sucrose into UDP-glucose and fructose (Taiz et al., 2014). Sorbitol dehydrogenase catalyzes the conversion of sorbitol and NAD^+^ into fructose and NADH + H^+^ (Nelson and Cox, 2005). Pullulanase, a starch debranching enzymes, can debranch pullulan and amylopectin (Fujita et al., 2009). α-1,4-Glucan phosphorylase L isozyme (spots 1′, 2′, 3′, and 4′), sucrose synthase 3 (spots 15′, 16′, and 17′), sorbitol dehydrogenase (spot 65′) and pullulanase (spots 5′, 6′, 7′, 8′, and 9′) abundances all decreased in the endosperm (Table 3, Supplementary Table S4, Figure 3B). We expect that the decreased abundance of these proteins is implicated in hybrid rice seed aging by decreasing the substrate supply to the embryo.

#### Seed aging is associated with an increased abundance of glycolytic and fermentation enzymes in the embryo

The important function of glycolysis and TCA cycle is to provide energy and carbon skeletons for cellular metabolism. In glycolysis, phosphoglucomutase converts glucose 1-phosphate to glucose 6-phosphate, triosephosphate isomerase catalyzes the reversible conversion of glyceraldehyde-3-phosphate (Gly-3-P) and dihydroxyacetone phosphate, Gly-3-P dehydrogenase catalyzes the conversion of Gly-3-P into 1,3-bisphosphoglycerate (BPGA), phosphoglycerate (PGA) kinase converts BPGA into 3-PGA, enolase converts 2-PGA to phosphoenolpyruvate, pyruvate kinase converts phosphoenolpyruvate to pyruvate (Taiz et al., 2014). Os03g0712700 (phosphoglucomutase, spot 13), triosephosphate isomerase (spots 65 and 66), Gly-3-P dehydrogenase 3 (spots 47 and 49), enolase (spot 32) and Os11g0148500 (pyruvate kinase 1, spot 24) abundances increased in embryos during aging of hybrid rice seeds (Table 3, Supplementary Table S3, Figure 3A). Consistent with this observation, Zhang et al. (2015) reported that triosephosphate isomerase, PGA kinase and enolase all increased in abundance in aged poplar seeds. Moreover, pyruvate decarboxylase 2 (spots 16, 17, 18, and 20) and alcohol dehydrogenase 1 (spot 42) increased in embryos (Table 3, Supplementary Table S3, Figure 3A). Pyruvate decarboxylase catalyzes the conversion of pyruvate into acetaldehyde, and alcohol dehydrogenase converts acetaldehyde into ethanol in fermentation (Taiz et al., 2014).

**Figure 5:**
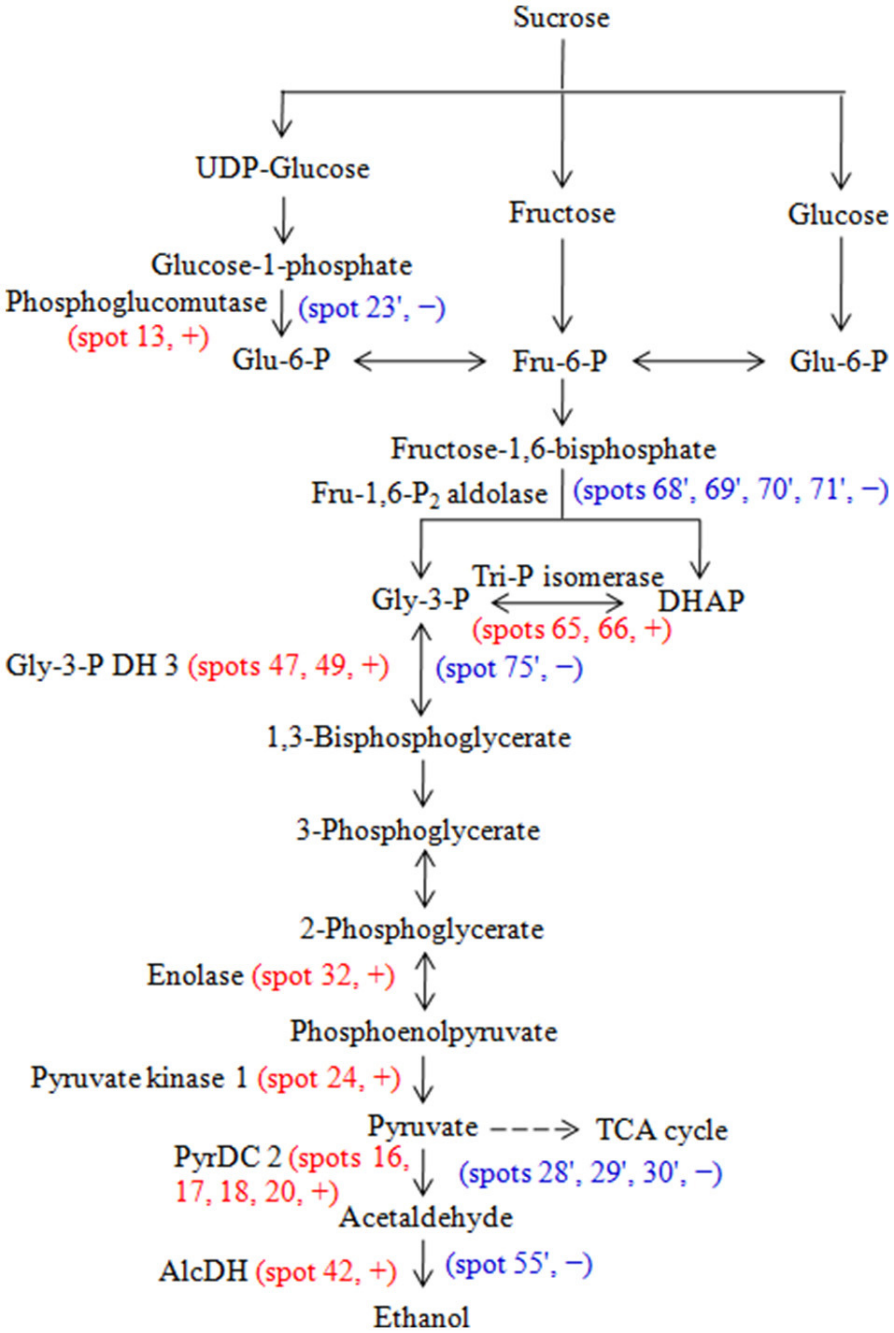
**Glycolysis and fermentation pathway and the enzymes that were identified as differentially changed proteins in embryos and endosperms during aging of Yliangyou 2 hybrid rice seeds**. Red and blue color indicates that the protein spot was identified in embryos and endosperms, respectively. +, increased; −, decreased; AlcDH, alcohol dehydrogenase; DHAP, dihydroxyacetone phosphate; Fru-6-P, fructose-6-phosphste; Fru-1,6-P_2_, fructose-1,6-bisphosphate; Glu-6-P, glucose-6-phosphate; Gly-3-P, glyceraldehyde-3-phosphate, Gly-3-P DH, Gly-3-P dehydrogenase; PyrDC, pyruvate decarboxylase; TCA, tricarboxylic acid; Tri-P, triose-phosphate.

We hypothesize that the following train of events can explain these observations: At high temperature and high humidity, the metabolic activity, including the rate of respiration, is high in the embryos. The result is hypoxia, which becomes so severe that mitochondrial respiration cannot function (Borisjuk and Rolletschek, 2009). To maintain an adequate ATP production the rate of glycolysis must be strongly increased, because glycolysis produces much less ATP per glucose molecule oxidized than respiration. And fermentation is induced in parallel (the Pasteur effect) to remove pyruvate, the end product of glycolysis. Therefore, the net result is that the end product of fermentation, ethanol, rapidly accumulates in the embryo during artificial aging leading eventually to toxic effects and decreased germination capability (Figure 5). Kodde et al. (2012) observed that ethanol accumulated during accelerated aging of *Brassica oleracea* seeds.

In the endosperm, many of the same glycolytic and fermentation enzymes decreased in abundance during seed aging. It is likely that the lower metabolic activity of the endosperm prevented the development of hypoxia and the induction of glycolysis and fermentation. The endosperm could export sugars to the embryo to help fuel its metabolism during aging.

#### Changes in abundance of proteins associated with protein synthesis and destination

##### Changes in abundance of proteins involved in protein synthesis

Aspartyl-tRNA synthetase catalyzes the formation of aspartyl-tRNA, an indispensable intermediate in protein biosynthesis (Nelson and Cox, 2005). Eukaryotic initiation factor 5A (eIF5A) is a highly conserved protein and contains two isoforms: eIF5A-1 and eIF5A-2. eIF5A promotes the formation of the first peptide bond at the initiation of protein synthesis (Wang et al., 2012b). During protein synthesis, elongation factor (EF)-Tu plays an important role in polypeptide elongation by promoting the GTP-dependent binding of aminoacyl-tRNA to the A site of the ribosome (Nelson and Cox, 2005). Hypothetical protein OsI_08509 (aspartyl-tRNA synthetase, spot 19), Os04g0118400 (elongation factor, spots 6 and 7) and elongation factor 1-gamma 3 (spot 39) abundances increased in embryos, while Os02g0686400 (putative aspartate-tRNA ligase, spot 32′), eukaryotic initiation factor 4A-1 (49′), eukaryotic translation initiation factor 5A-2 (spot 93′) and Os02g0519900 (elongation factor 2, spot 13′) all decreased in endosperms (Table 3, Supplementary Tables S3, S4, Figure 3).

##### Changes in abundance of proteins involved in proteolysis

The ubiquitin–proteasome system is responsible for the elimination of misfolded and damaged, potentially toxic, proteins produced in response to different cellular stresses. The degradation of proteins by the ubiquitin–proteasome system includes two steps. The first step is the covalent conjugation of ubiquitin to the targeted protein, the second is degradation of the ubiquitin-tagged protein by the 26S proteasome (Sankiewicz et al., 2015). Os05g0187000 (proteasome subunit beta type, spot 67) increased in embryos (Table 3, Supplementary Table S3, Figure 3A).

Leucine aminopeptidase (LAP) is ubiquitously found in all living organisms. Arabidopsis LAP2, an enzymatically active aminopeptidase, is responsible for the cleavage of leucine, methionine and phenylalanine from *N*-terminal peptides, and plays important roles in various cellular processes in plants (Waditee-Sirisattha et al., 2011). The Clp protease family is conserved among eubacteria and most eukaryotes, and uses ATP to drive protein substrate unfolding and translocation into the active sites. The main constitutive Clp protease in photosynthetic organisms has evolved into a functionally essential and structurally intricate enzyme (Andersson et al., 2009). LAP2 (spot 40′) and Os02g0634500 (ATP-dependent Clp protease proteolytic subunit, spot 83′) decreased in endosperms (Table 3, Supplementary Table S4, Figure 3B).

#### Increased storage protein degradation is associated with hybrid rice seed aging

Storage proteins are mainly synthesized during the late stage of seed development and deposited in protein storage vacuoles in dried mature seeds. They may supply energy and amino acids for subsequent seed germination and seedling growth (Bewley et al., 2013). Endosperm lumenal binding protein (spot 20′), Os05g0116000 (putative legumin, spot 77′), putative globulin (with alternative splicing, spot 79′), globulin-like protein (spot 96′) and seed allergenic protein RAG2 (spot 95′) all decreased in abundance in endosperms during hybrid rice seed aging (Table 3, Supplementary Table S4, Figure 3B).

We observed that putative globulin (with alternative splicing, spots 29, 30, and 56), Os03g0663800 (putative globulin, with alternative splicing, spots 57 and 58), globulin-like protein (spots 71 and 75), and hypothetical protein OsI_13867 (globulin-like protein, spots 38, 51, 53, and 64) increased in abundance in embryos during hybrid rice seed aging (Table 3, Supplementary Table S3, Figure 3A). With one exception, the experimental size of these proteins was much smaller than their theoretical size (Table 1), indicating that they derived from protein degradation. The exception was hypothetical protein OsI_13867 (globulin-like protein, spot 38), which appeared at a slightly larger size and a slightly lower pI (Table 1), probably caused by a posttranslational modification other than proteolytic degradation. These results reveal that degradation of storage proteins in embryos and endosperms was closely linked to aging of hybrid rice seeds. Ching and Schoolcraft (1968) predicted that the content of seed storage protein would decrease upon seed aging. Xin et al. (2011) reported that vicilin-like storage protein and globulin 2 decreased in maize embryos during seed aging. Recently, Nguyen et al. (2015) proposed that oxidation is involved in seed deterioration and that seed storage proteins buffer the seed from oxidative stress, thus protecting important proteins required for seed germination and seedling formation. This is consistent with the changes in proteolytic enzymes discussed above.

#### Changes in protein involved in cell defense and rescue

##### Change in abundance of defense-related proteins

Cysteine proteinase inhibitors (CysPIs) are ubiquitously distributed among animals, plants and microorganisms, and specifically inhibit sulfhydryl proteinases. Physiological functions of CysPIs are regulation of protein turnover and host plant defense against insect predation and, perhaps, pathogens (Zhao et al., 1996). CysPI 12 (spot 69) increased in abundance in embryos (Table 3, Supplementary Table S3, Figure 3A), which was consistent with the observation by Zhang et al. (2015), who found that CysPI increased in low vigor poplar seeds.

Most members of the serpin family of proteins are potent, irreversible inhibitors of specific serine or cysteine proteinases. Inhibitory serpins can be distinguished from members of other families of proteinase inhibitors by their metastable structure and unique suicide-substrate mechanism (Francis et al., 2012). We observed that serpin-ZXA (spot 62′) abundance decreased in the endosperms during hybrid rice seed aging (Table 3, Supplementary Table S4, Figure 3B).

##### Change in abundance of proteins involved in detoxification

ROS are the main contributors to hybrid rice seed aging (Yin et al., 2014), by causing lipid and protein oxidation and DNA damage (Møller et al., 2007, 2011). Superoxide dismutase (SOD), an enzyme induced by many stress factors, converts ${\text{O}}_{2}^{\cdot -}$ to H_2_O_2_, dehydroascorbate reductase (DHAR) converts oxidized ascorbate into reduced ascorbate, and they therefore both play important roles in ROS detoxification (Cheng and Song, 2008). SOD (spot 82) increased in embryos, while Os05g0116100 (DHAR, spots 84′ and 85′) decreased in endosperms (Table 3, Supplementary Tables S3, S4, Figure 3).

Methylglyoxal, a byproduct of normal biochemistry in nature, is highly toxic because of its chemical reactions with proteins, nucleic acids, and other cellular components. Lactoylglutathione lyase (known as glyoxalase I), an enzyme in methylglyoxal detoxification, converts glutathione and methylglyoxal into S-D-lactoylglutathione (Rabbani et al., 2014). Aldehyde dehydrogenases (ALDHs), a protein superfamily encoding NAD(P)^+^-dependent enzymes, oxidize a wide range of endogenous and exogenous aliphatic and aromatic aldehydes. They are involved in many biological processes and play a role in the response to environmental stress in plant (Li et al., 2013). Lactoylglutathione lyase (spots 55 and 60) and aldehyde dehydrogenase (spot 31) increased in abundance in embryos during seed aging (Table 3, Supplementary Table S3, Figure 3A).

Most annexins are Ca^2+^-dependent, phospholipid-binding proteins with unclear functions in response to environmental stresses and signaling during plant growth and development (Jami et al., 2012). Hypothetical protein OsI_08976 (annexin, spot 54) increased in embryos (Table 3, Supplementary Table S3, Figure 3A), which may be involved in hybrid rice seed aging.

##### Change in abundance of proteins involved in stress response

Molecular chaperones are proteins that assist the covalent folding or unfolding and the assembly or disassembly of other macromolecular structures (Ellis, 2006). Singh et al. (2010) reported that chaperone protein ClpB1 was expressed in developing embryos and dry seeds, but decreased rapidly during seed germination. Chaperone protein ClpB1 (spots 1 and 2) increased in abundance in embryos during seed aging (Table 3, Supplementary Table S3, Figure 3A).

Heat shock cognate 70 kDa protein (spot 12) increased in embryos (Table 3, Supplementary Table S3, Figure 3A) is consistent with the observation by Zhang et al. (2015), who found that heat shock factor binding proteins increased in abundance in low vigor poplar seeds.

LEA protein 1 (spots 43 and 44) and hypothetical protein OsI_06577 (putative LEA protein, spots 27 and 28) increased in embryos during seed aging (Table 3, Supplementary Table S3, Figure 3A). We also observed that HSP 70 (spot 22′), heat shock cognate 70 kDa protein (spot 21′), Os02g0644100 (putative stress-induced protein sti1, spot 24′) and LEA protein group 3 (spot 86′) decreased in endosperms during seed aging (Table 3, Supplementary Table S4, Figure 3B).

These observations all indicate that there is an increasing need for protein stabilization during hybrid rice seed aging.

## Conclusions

The aging of hybrid rice seeds is a quantitative trait, as germination percentage and germination rate gradually decreased with increasing aging time. By monitoring changes in proteome of embryo and endosperm from seeds aged for different duration, it is possible to form a picture of the events that lead to the loss of germinability: A general observation was that many endosperm proteins decreased in abundance during aging. To the extent that this required changes in gene expression, the signals must have come from the aleurone layer and/or the embryo. Another significant observation was the increased abundance of glycolytic and fermentation enzymes in the embryo, but not in the endosperm (Figure 5), which indicates that fermentation has taken the place of respiration in providing energy for the embryo. This shift must have been caused by hypoxia, which is consistent with the observations that several enzymes involved in ROS detoxification, proteins involved in stress response, such as protein stabilization, and proteolytic enzymes, possibly involved in removal of damaged proteins, all increased in the embryo, but not in the endosperm. The end product of fermentation is ethanol and the embryos may be gradually poisoned by accumulating ethanol during artificial aging.

## Author contributions

YZ, HX, SS, and IMM designed the experiments and YZ, HX, SL, NL, WW, and SS performed them. HX, SS, and IMM analyzed the results. And HX, SS, and IMM wrote the paper.

### Conflict of interest statement

The authors declare that the research was conducted in the absence of any commercial or financial relationships that could be construed as a potential conflict of interest.

## Supplementary material

Supplementary Figure S1 **The gel maps of total proteins from embryos of Yliangyou 2 hybrid rice seeds aged in 100% RH and at 40°C for (A) 0, (B) 10, and (C) 25 days**. After CBB staining, 1109 ± 103 protein spots were detected, which is a mean ± SD of protein spots of different treatments. −1, −2, and −3 represent three independent biological replicates.

Supplementary Figure S2 **The gel maps of total proteins from endosperms of Yliangyou 2 hybrid rice seeds aged in 100% RH and at 40°C for (A) 0, (B) 10, and (C) 25 days**. After CBB staining, 1093 ± 93 protein spots were detected, which is a mean ± SD of protein spots of different treatments. −1, −2, and −3 represent three independent biological replicates.

Supplementary Table S1 **The MS/MS details of the identified proteins by MALDI-TOF-TOF MS in embryos during aging of Yliangyou 2 hybrid rice seeds**.

Supplementary Table S2 **The MS/MS details of the identified proteins by MALDI-TOF-TOF MS in endosperms during aging of Yliangyou 2 hybrid rice seeds**.

Supplementary Table S3 **Accumulation levels, accumulation ratios and associated *P*-values for proteins changing in abundance and identified by MALDI-TOF-TOF MS in embryos during aging of Yliangyou 2 hybrid rice seeds**.

Supplementary Table S4 **Accumulation levels, accumulation ratios and associated *P*-values for proteins changing in abundance and identified by MALDI-TOF-TOF MS in endosperms during aging of Yliangyou 2 hybrid rice seeds**.

Supplementary Table S5 **The number of protein spots changing in abundance and identified in embryos during aging of Yliangyou 2 hybrid rice seeds**.

Supplementary Table S6 **The number of protein spots changing in abundance and identified in endosperms during aging of Yliangyou 2 hybrid rice seeds**.

Supplementary Table S7 **Protein spots with two or more than two proteins in embryos during aging of Yliangyou 2 hybrid rice seeds**.

Supplementary Table S8 **Protein spots with two or more than two proteins in endosperms during aging of Yliangyou 2 hybrid rice seeds**.
