# Supplementary material for: Proteomic Analysis Reveals Different Involvement of Embryo and Endosperm Proteins during Aging of Yliangyou 2 Hybrid Rice Seeds
Source: Front Plant Sci. 2016 Sep 21;7:1394. doi: 10.3389/fpls.2016.01394 (PMC5031166; doi:10.3389/fpls.2016.01394)
Supplement: Supplementary Table S5 — The number of protein spots changing in abundance and identified in embryos during aging of Yliangyou 2 hybrid rice seeds. [file Table5.DOC]

**Supplementary Table S5 │** The number of protein spots changing in abundance and identified in embryos during ageing of Yliangyou 2 hybrid rice seeds.

|  | **Total**  **number** |
| --- | --- |
| Total number of protein spots changed differentially (≥2.0-fold change and *P*<0.05) | 91 |
| Unidentified protein spots | 16 |
| Identified protein spots | 75 |
| Protein spots with only one identified protein (Table 1, Supplementary Table S1) | 71 |
| Proteins spots with two or three identified proteins (Supplementary Table S5) | 4 |
