# Supplementary material for: Proteomic Analysis Reveals Different Involvement of Embryo and Endosperm Proteins during Aging of Yliangyou 2 Hybrid Rice Seeds
Source: Front Plant Sci. 2016 Sep 21;7:1394. doi: 10.3389/fpls.2016.01394 (PMC5031166; doi:10.3389/fpls.2016.01394)
Supplement: Supplementary Table S6 — The number of protein spots changing in abundance and identified in endosperms during aging of Yliangyou 2 hybrid rice seeds. [file Table6.DOC]

**Supplementary Table S6** **│** The number of protein spots changing in abundance and identified in endosperms during ageing of Yliangyou 2 hybrid rice seeds.

|  | **Total**  **number** |
| --- | --- |
| Total number of protein spots changed differentially (≥2.0-fold change and *P*<0.05) | 100 |
| Unidentified protein spots | 8 |
| Identified protein spots | 92 |
| Protein spots with only one identified protein (Table 2, Supplementary Table S2) | 79 |
| Protein spots with two or more than two identified proteins (Supplementary Table S6) | 13 |
