# Supplementary material for: Proteomic Analysis Reveals Different Involvement of Embryo and Endosperm Proteins during Aging of Yliangyou 2 Hybrid Rice Seeds
Source: Front Plant Sci. 2016 Sep 21;7:1394. doi: 10.3389/fpls.2016.01394 (PMC5031166; doi:10.3389/fpls.2016.01394)
Supplement: Supplementary Table S7 — Protein spots with two or more than two proteins in embryos during aging of Yliangyou 2 hybrid rice seeds. [file Table7.DOC]

**Supplementary Table S7** │ Protein spots with two or more than two proteins in embryos during ageing of Yliangyou 2 hybrid rice seeds.

| **Spot ID** | **Identified protein** | **Accession No.** | **Mascot score** | **Sequence coverage (%)** | **No. of sequenced /matched peptides** | **Theoretical protein mass (kDa) /pI** | **Experimental protein mass (kDa) /pI** | **Biological processes** |
| --- | --- | --- | --- | --- | --- | --- | --- | --- |
| 3 | Putative aconitate hydratase, cytoplasmic | Q6YZX6 | 464 | 34 | 9/25 | 98.591/5.67 | 76/6.1 | Energy |
|  | Chaperone protein ClpB2, chloroplastic | Q75GT3 | 159 | 22 | 4/20 | 109.146/6.25 | 76/6.1 | Cell defense and rescue |
| 15 | Hypothetical protein OsI_03698 (malic enzyme) | EAY75782 | 561 | 32 | 8/18 | 63.578/7.11 | 64/7.0 | Metabolism |
|  | Hypothetical protein OsI_13867 (globulin-like protein)* | EEC76319 | 104 | 15 | 3/5 | 52.370/6.99 | 64/7.0 | Storage protein |
| 23 | Hypothetical protein OsI_24243 (T-complex protein 1 subunit eta)* | EEC81211 | 273 | 22 | 3/7 | 60.228/6.31 | 60/6.5 | Protein synthesis and destination |
|  | Os06g0247500 (putative pyrophosphate-dependent phosphofructokinase beta subunit) | NP_001057284 | 199 | 14 | 3/7 | 61.907/6.01 | 60/6.5 | Energy |
|  | Cysteinyl-tRNA synthetase, putative, expressed | ABF93928 | 141 | 16 | 3/5 | 39.234/6.15 | 60/6.6 | Protein synthesis and destination |
| 37 | Hypothetical protein OsI_13867 (globulin-like protein)* | EEC76319 | 740 | 55 | 10/24 | 52.370/6.99 | 54/6.9 | Storage protein |
|  | OSIGBa0124N08.2 (ATP-dependent 6-phosphofructokinase) | CAH67040 | 138 | 36 | 3/15 | 51.543/6.23 | 54/6.9 | Energy |
