# Supplementary material for: Proteomic Analysis Reveals Different Involvement of Embryo and Endosperm Proteins during Aging of Yliangyou 2 Hybrid Rice Seeds
Source: Front Plant Sci. 2016 Sep 21;7:1394. doi: 10.3389/fpls.2016.01394 (PMC5031166; doi:10.3389/fpls.2016.01394)
Supplement: Supplementary Table S8 — Protein spots with two or more than two proteins in endosperms during aging of Yliangyou 2 hybrid rice seeds. [file Table8.DOC]

**Supplementary Table S8** │ Protein spots with two or more than two proteins in endosperms during ageing of Yliangyou 2 hybrid rice seeds.

| **Spot ID** | **Identified protein** | **Accession No.** | **Mascot score** | **Sequence coverage (%)** | **No. of sequenced /matched peptides** | **Theoretical protein mass (kDa)/pI** | **Experimental protein mass (kDa) /pI** | **Biological processes** |
| --- | --- | --- | --- | --- | --- | --- | --- | --- |
| 27' | Hypothetical protein OsI_03698 (malic enzyme) | EAY75782 | 569 | 38 | 9/21 | 63.578/7.11 | 62/7.8 | Metabolism |
|  | Putative globulin (with alternative splicing) | AAS07324 | 413 | 29 | 6/12 | 63.845/8.35 | 62/7.8 | Storage protein |
| 33' | Os06g0114000 (rubisco subunit binding-protein beta subunit) | NP_001056601 | 1010 | 55 | 11/28 | 64.331/5.60 | 67/5.5 | Protein synthesis and destination |
|  | Glucose-6-phosphate isomerase | BAD08451 | 230 | 34 | 3/17 | 68.765/5.88 | 67/5.5 | Energy |
| 34' | Os06g0247500 (putative pyrophosphate-dependent phosphofructokinase beta subunit) | NP_001057284 | 646 | 66 | 9/26 | 61.907/6.01 | 66/6.5 | Energy |
|  | Os08g0559600 (putative dihydroxy-acid dehydratase) | NP_001062503 | 192 | 22 | 4/9 | 64.237/7.12 | 66/6.5 | Metabolism |
| 41' | Putative selenium binding protein | BAB40923 | 465 | 53 | 7/17 | 51.325/5.73 | 63/6.2 | Cell defense and rescue |
|  | Serine hydroxymethyltransferase, mitochondrial precursor, putative, expressed | ABA97575 | 193 | 27 | 6/13 | 58.569/6.61 | 63/6.2 | Metabolism |
| 45' | Aldehyde dehydrogenase | AAF73828 | 313 | 31 | 7/14 | 59.626/6.33 | 62/6.2 | Cell defense and rescue |
|  | UDP-glucose pyrophosphorylase | ABD57308 | 130 | 24 | 3/8 | 51.791/5.43 | 62/6.2 | Metabolism |
|  | Putative alanine aminotransferase | AAK52114 | 117 | 16 | 3/7 | 53.229/6.23 | 62/6.2 | Metabolism |
|  | Metalloenzyme superfamily protein, expressed | ABA98832 | 105 | 21 | 3/8 | 48.303/5.52 | 62/6.2 | Cell defense and rescue |
| 46' | Aldehyde dehydrogenase | AAF73828 | 879 | 50 | 9/21 | 59.626/6.33 | 61/6.3 | Cell defense and rescue |
|  | Putative alanine aminotransferase |  | 196 | 19 | 3/7 | 53.229/6.23 | 61/6.3 | Metabolism |
| 58' | Hypothetical protein OsI_10172 (embryonic protein DC-8 precursor)* | EAY88696 | 223 | 19 | 3/8 | 39.933/8.29 | 52/8.0 | Unknown |
|  | Hypothetical protein OsI_26662 (α-L-fucosidase 2 precursor)* | EAZ04511 | 143 | 14 | 3/5 | 42.995/7.49 | 52/8.0 | Metabolism |
| 66' | Hypothetical protein OsI_30128 (sorbitol dehydrogenase)* | EEC83981 | 691 | 66 | 8/19 | 39.184/5.97 | 49/6.7 | Metabolism |
|  | Putative serine protease inhibitor | AAR87358 | 68 | 13 | 3/6 | 70.747/5.88 | 49/6.7 | Cell defence and rescue |
| 67' | Arginase 1, mitochondrial | B8AU84 | 434 | 67 | 5/16 | 37.179/5.90 | 48/6.0 | Protein synthesis and destination |
|  | Uroporphyrinogen decarboxylase 2 | Q10LR9 | 60 | 10 | 3/4 | 43.045/6.15 | 48/6.0 | Metabolism |
| 78' | Unknown protein (scaffold attachment factor)* | AAN05517 | 796 | 67 | 9/16 | 35.437/5.57 | 39/6.1 | Unknown |
|  | Os01g0850900 (SOUL heme-binding protein-like) | NP_001044817 | 177 | 24 | 3/6 | 23.588/5.87 | 39/6.1 | Metabolism |
| 80' | Os04g0404400 (desiccation-related protein PCC13-62)* | BAS89067 | 523 | 48 | 6/11 | 32.258/4.91 | 36/5.6 | Cell defence and rescue |
|  | Cupin family protein, expressed | ABF95817 | 422 | 27 | 6/13 | 61.742/7.18 | 36/5.6 | Storage protein |
| 81' | Os12g0169700 (γ-carbonic anhydrase 1, CA1)* | NP_001066265 | 317 | 47 | 4/9 | 29.670/6.42 | 35/7.3 | Energy |
|  | Os03g0822200 (NAD-dependent epimerase/dehydratase)* | NP_001051733 | 114 | 38 | 3/8 | 27.950/6.34 | 35/7.3 | Unknown |
| 94' | Os03g0305600 (mitochondrial import inner membrane translocase subunit Tim17/Tim22/Tim23 family protein, putative, expressed) | NP_001049884 | 406 | 38 | 5/8 | 18.416/6.42 | 20/6.4 | Transport |
|  | Os07g0597000 (eukaryotic translation initiation factor 5A) | NP_001060174 | 279 | 43 | 3/6 | 17.675/5.77 | 20/6.4 | Protein synthesis and destination |
